# Endoluminal radiofrequency ablation for ingrowth occlusion after endoscopic ultrasound-guided hepaticogastrostomy with bridging stent placement

**DOI:** 10.1055/a-2717-1332

**Published:** 2025-10-21

**Authors:** Kenjiro Yamamoto, Takayoshi Tsuchiya, Ryosuke Tonozuka, Shuntaro Mukai, Hiroyuki Kojima, Noriyuki Hirakawa, Takao Itoi

**Affiliations:** 1 Department of Gastroenterology and Hepatology, Tokyo Medical University, Tokyo, Japan

Endoscopic ultrasound-guided hepaticogastrostomy with bridging stent placement is a useful drainage option when endoscopic retrograde cholangiopancreatography fails for reasons such as inaccessible papillae or surgical anastomosis, particularly in patients with malignant hilar biliary obstruction who require bilateral drainage 1 2 . Recent advances in chemotherapy, such as targeted drugs for specific gene mutations, have improved prognosis in biliary tract cancers 3 . Therefore, the management of recurrent biliary obstruction after stent placement is also an important issue. Endoluminal radiofrequency ablation (RFA) is a novel procedure for biliary diseases and may be an option for treating ingrowth occlusion after metal stent deployment 4 5 .

An 80-year-old man was admitted with obstructive jaundice during chemotherapy for local relapse after Whipple surgery for cholangiocarcinoma. He had previously undergone right hepatic drainage with bridging metal stent placement through the hepaticogastrostomy route. Contrast-enhanced computed tomography demonstrated dilatation of the anterior bile duct ( Fig. 1 ). Cholangiography and peroral cholangioscopy (POCS) revealed stent occlusion due to tumor ingrowth ( Fig. 2 ). Additional stent placement through previously inserted bilateral metal stents was considered technically challenging. Thus, RFA was performed with a temperature-controlled RF catheter (ELRA; StarMed Co., Goyang, Korea). The RF generator (VIVA Combo; StarMed Co.) was set to a maximum temperature of 80°C and power of 7W for a 2-min duration and was then connected to an 18-mm RF catheter. Ablation was performed stepwise, spanning the stricture ( Fig. 3 ). After RFA, a temporary endoscopic nasobiliary drainage tube was placed. Four days after RFA, cholangiography and POCS confirmed ablation of the ingrowth regions and recanalization of the stent lumen ( Fig. 4 ; Video 1 ). The patient was discharged after plastic stent placement through the hepaticogastrostomy route. Recanalization using RFA was clinically successful, with no recurrence of biliary obstruction for more than 3 months.

**Fig. 1:**
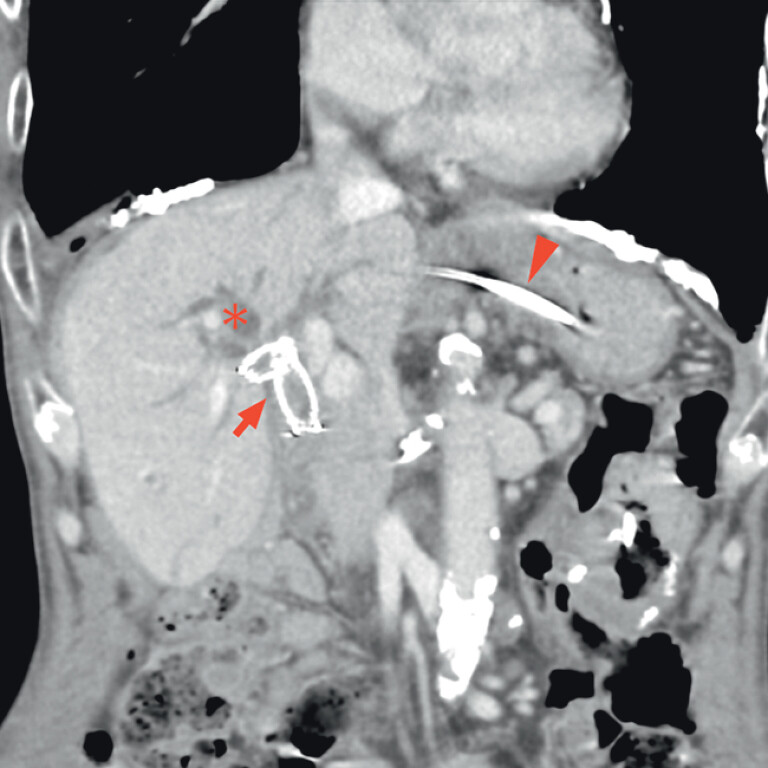
Contrast-enhanced computed tomography showing dilatation of the anterior bile duct (asterisk). For local relapse after Whipple surgery for cholangiocarcinoma, right hepatic drainage with bridging metal stent placement through the hepaticogastrostomy route had previously been performed and another metal stent placed from the jejunum to the left hepatic duct in a stent-in-stent configuration (arrow). A plastic stent had been placed across the hepaticogastrostomy route (arrowhead).

**Fig. 2:**
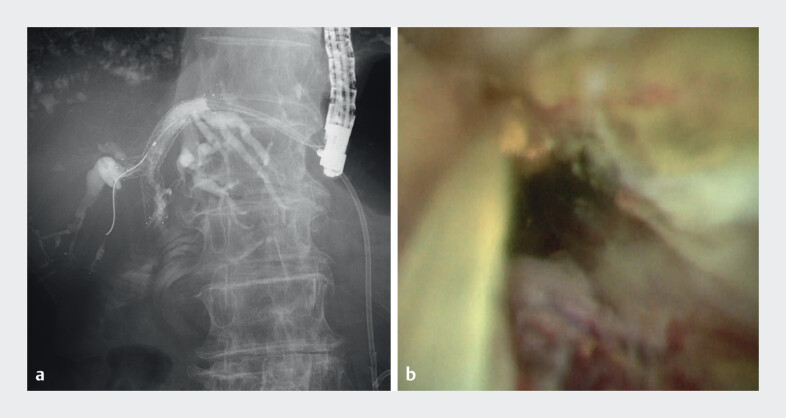
Stent occlusion due to tumor ingrowth: **a** cholangiographic view; **b** peroral cholangioscopic view.

**Fig. 3:**
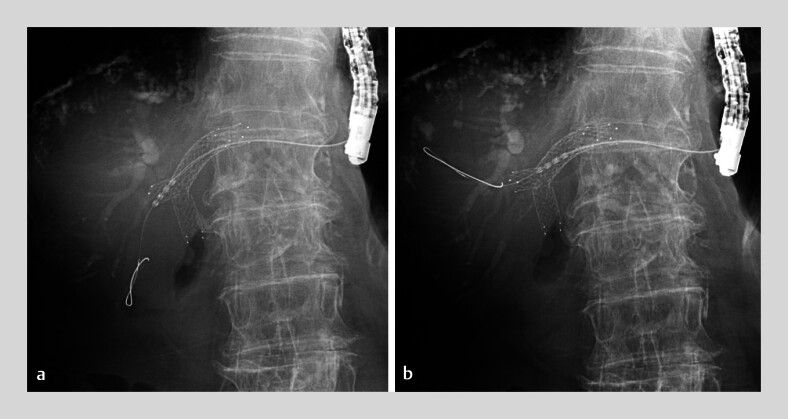
Stepwise endoluminal radiofrequency ablation spanning the stricture: fluoroscopic view.

**Fig. 4:**
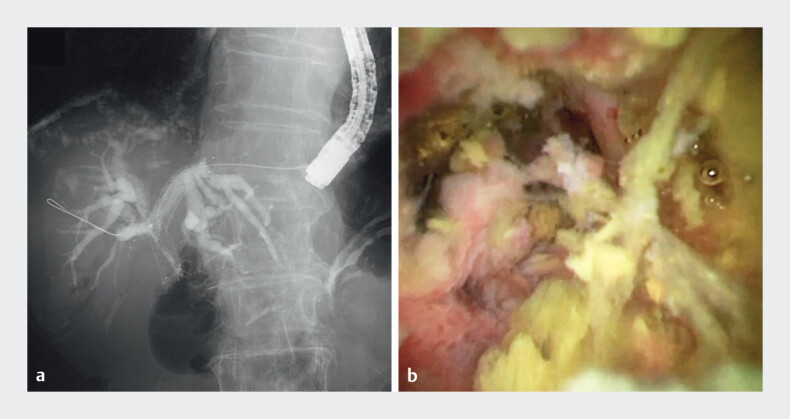
Successful ablation of the ingrowth regions and recanalization of the stent lumen: **a** cholangiographic view; **b** peroral cholangioscopy.

Radiofrequency ablation for ingrowth occlusion after endoscopic ultrasound-guided hepaticogastrostomy with bridging stent placement.Video 1

Endoscopy_UCTN_Code_TTT_1AR_2AZ

## Notes

Endoscopy E-Videos https://eref.thieme.de/e-videos
*E-Videos* is an open access online section of the journal *Endoscopy* , reporting on interesting cases and new techniques in gastroenterological endoscopy. All papers include a high-quality video and are published with a Creative Commons CC-BY license. Endoscopy E-Videos qualify for HINARI discounts and waivers and eligibility is automatically checked during the submission process. We grant 100% waivers to articles whose corresponding authors are based in Group A countries and 50% waivers to those who are based in Group B countries as classified by Research4Life (see: https://www.research4life.org/access/eligibility/ ). This section has its own submission website at https://mc.manuscriptcentral.com/e-videos .
